# Knowledge, Attitude, and Beliefs of Communities and Health Staff about* Echinococcus granulosus* Infection in Selected Pastoral and Agropastoral Regions of Uganda

**DOI:** 10.1155/2018/5819545

**Published:** 2018-05-13

**Authors:** Emmanuel Othieno, Michael Ocaido, Ezekiel Mupere, Leonard Omadang, Peter Oba, Andrew Livex Okwi

**Affiliations:** ^1^Department of Pathology, School of Biomedical Sciences, College of Health Sciences, Makerere University, P.O. Box 7072, Kampala, Uganda; ^2^Department of Wild Life, School of Veterinary Medicine and Animal Resources, College of Veterinary Medicine, Animal Resources and Biosecurity, Makerere University, P.O. Box 7062, Kampala, Uganda; ^3^Department of Pediatrics, School of Medicine, College of Health Sciences, Makerere University, P.O. Box 7072, Kampala, Uganda; ^4^National Agricultural Research Organization, Abi Zonal Agricultural Research and Development Institute, P.O. Box 219, Arua, Uganda

## Abstract

A descriptive cross-sectional survey was done to determine knowledge, attitudes, and beliefs of the communities and health workers about cystic echinococcosis (CE) in pastoral region of Northeastern (NE) and agropastoral regions of Eastern (E) and Central (C) Uganda. Overall a total of 1310 participants were interviewed. Community respondents from NE region were more aware of CE infection than those from Eastern (OR 4.85; CI: 3.60–6.60; *p* < 0.001) and Central (OR 5.73; CI: 4.22–7.82; *p* < 0.001) regions. 19.8% of the respondents from EA region had positive attitude towards visiting witch doctors for treatment compared with 62.0% and 60.4% from NE and Central regions, respectively (*p* < 0.001). Notably, the awareness of CE increased with level of education (*p* < 0.001). There was no statistical difference between male and female respondents as far as awareness of CE was concerned (*p* > 0.05). 51.7% of the community respondents from Central believed CE is caused by witchcraft, compared with 31.3% and 14.3% from NE and EA regions, respectively (*p* < 0.001). There was no statistical difference between health staff regarding their knowledge, attitude, and beliefs about CE infection (*p* > 0.05). None of the participants knew his/her CE status. The communities need to be sensitized about CE detection, control, and management and health staff need to be trained on CE diagnosis.

## 1. Introduction

According to World Health Organization (WHO) [1] cystic echinococcosis (CE) is a neglected zoonotic infection found throughout the world and is associated with high morbidity and mortality in poor resource countries especially in pastoral communities in Africa (Macpherson et al. [2]). In Uganda, the prevalence of CE has been found to vary between pastoral and agropastoral communities, with pastoral communities being at higher risk than agropastoral communities (Othieno et al. [3]). High prevalence of CE has equally been reported in livestock (Chamai et al. [4] and Magambo et al. [5]) and in dogs (Inangolet et al. [6] and Oba et al. [7]). Cystic echinococcosis is caused by a species of* Echinococcus,* namely,* Echinococcus granulosus,* whose definitive hosts are the carnivores such as dogs. Usually dogs become infected with* Echinococcus granulosus* by eating infected internal organs such as liver and lungs from dead animals that contain tape worm embryos. The dogs pass out tapeworm eggs in their stool, which can cause infection in other animals and/or in humans who accidentally swallow the eggs. In humans,* Echinococcus granulosus* forms slow-growing cysts (called hydatid cysts) in different organs of the body which can be very difficult to remove or treat in some cases (Nahmias et al. [8]).

Increased awareness of zoonotic infections has been found to influence the management and control of these diseases. However, lack of adequate knowledge by the communities on echinococcosis transmission has been linked to wide spread of the disease within and outside the communities in sub-Saharan African countries (John et al. [9]). Similarly, lack of knowledge by health staff on the diagnosis and treatment of CE has been found to be associated with poor management and control of the disease (Reyes et al. [10]). This has therefore contributed to underdiagnosis and reporting of zoonotic diseases thus culminating into poor disease monitoring coverage and lack of clear interventions to address the burden of zoonotic diseases (Reyes et al. [10]). An adequate information on knowledge, attitudes, and beliefs about echinococcosis by communities is therefore vital for them to play an important public health role (Otupiri et al. [11]). In addition, training of the health workers on the use of ultrasound for early diagnosis of CE is paramount. In Uganda, studies on the knowledge, beliefs, and attitudes of the communities and health workers about CE are scanty. It was against this background that this study was designed to determine the knowledge, attitudes, and beliefs of communities and health staff about echinococcosis infection in selected pastoral and agropastoral regions of Uganda.

## 2. Materials and Methods

### 2.1. Study Design

This was a descriptive cross-sectional survey conducted from July 2012 to January 2014.

### 2.2. Setting

**Figure 1:**
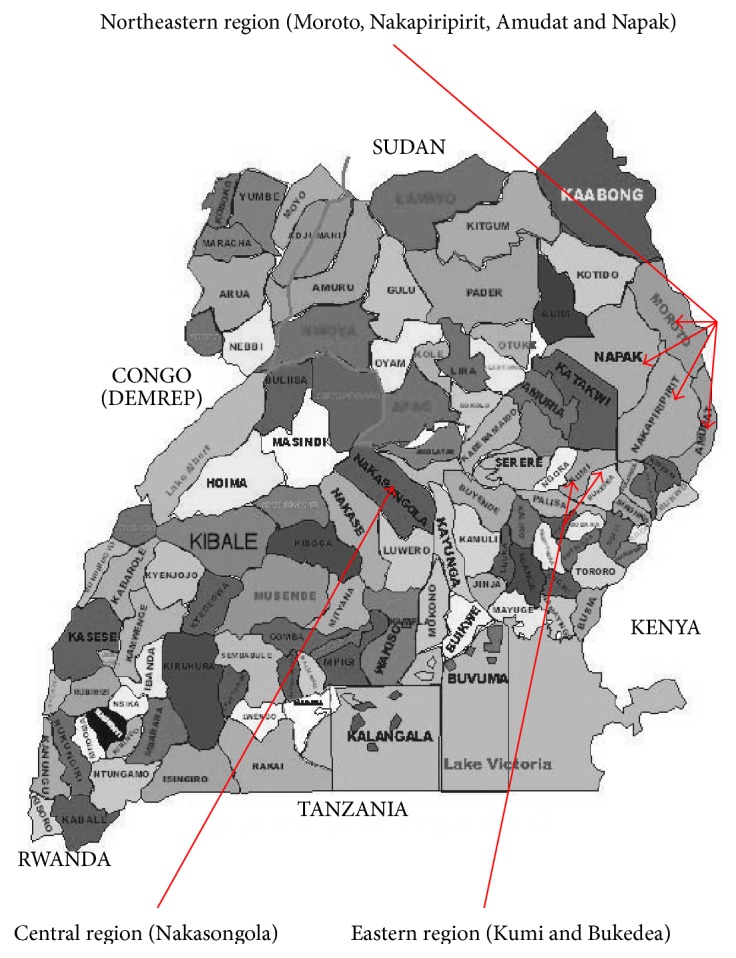
Map of Uganda showing the selected study regions.

The study comprised pastoral region of Northeastern and agropastoral regions of Eastern and Central Uganda. The districts of Nakapiripirit, Amudat, Moroto, and Napak were randomly selected in Northeastern region, while the districts of Kumi and Bukedea were selected in Eastern region. Nakasongola district was selected in Central region. The details of the regions is as shown in Figure 1 [12].

The selection of these regions was based on the predominance of the pastoral production system (Karamoja subregion) or mixed crop-livestock production systems (Eastern and Central subregions), where there is a high prevalence of CE in humans (Magambo et al. [5]), livestock (Chamai et al. [4]), and dogs as previously reported (Inangolet et al. [6] and Oba et al. [7]). These are remote, hard to reach communities with poor health infrastructure and with no specific control programs for CE.

### 2.3. Study Population

It comprised communities and health staff. Only the nurses and paramedical staff from Health Centers IVs were identified to avoid bias because they all had the same level of education background.

The prevalence of 66.3% of echinococcosis which was found in dogs (Inangolet et al. [6]) was used for the determination of the sample size for KAPs. It was assumed that the prevalence of echinococcosis in dogs would reflect the same prevalence of echinococcosis in humans, since the dogs are the primary hosts. The sample size calculation was then done using the equation of Kish and Leshlie (Kirkwood [13]) for proportions in cross-sectional studies.

*n* = (*Z*^2^/*d*^2^)*PQ*, where *Z* is the value of 1.96 (*Z* in normal distribution curve), *n* is the required sample size, *p* is the estimated prevalence of CE, *Q* = 100 − *P*, and *d* is the required precision (5%). Using this equation, a total sample size of 1,200 individuals in all the regions was therefore computed. However, we interviewed a total of 1,235 respondents.

### 2.4. Data Collection Procedure

Pretested structured questionnaires were used to generate information from eligible participants. Community participants were conveniently mobilized with the assistance of the elders and local leaders and brought to trading centers which had been identified for interviews. Random sampling procedure was then used to select community respondents. The names of the respondents were written in small chits of paper and then folded. Names of those to be interviewed were then randomly picked. The health staffs were consecutively recruited from their health facilities. Participation was limited to those voluntarily willing to take part in the study. All the participants were interviewed after seeking their consent.

### 2.5. Data Analysis

The data were entered and analyzed using software package for social sciences 10.0 (SPSS 10.0) [14]. The statistical differences between respondents on the knowledge, attitude, and beliefs about echinococcosis were compared using open source epidemiologic statistic soft ware program for public health version 2.2.1 (OPENEPI) using 2 × 2 contingency tables [15]. Odds ratios and 95% confidence intervals were computed. A *p* value of 0.05 was considered statistically significant.

## 3. Results

### 3.1. Sociodemographic Characteristics (Distribution) of Respondents

A total of 421 respondents were identified and interviewed in Northeastern region, 405 from Eastern region, and 409 from Central region, giving a total of 1,235 respondents, which was 2.9% a little more than the calculated sample size of 1,200. A total of 75 health workers were interviewed in all the regions giving an overall total of 1310 participants. A total of 720 males and 590 females were interviewed. 291 respondents from Northeastern region had informal education, 167 from Eastern region, and 187 from Central region. Their ages ranged between 18 and 80 years giving mean age of 49 years. The details of the sociodemographic characteristics are as shown in Table 1.

### 3.2. Community Knowledge about CE

The results showed that 60.8% of the respondents in Northeastern region (NE) were aware of CE infection compared with 24.2% in Eastern (OR 4.9, CI: 2.58–9.57, and *p* < 0.001) and 21.3% in Central regions (OR 5.8, CI: 3.0–11.6, and *p* < 0.001). A significant difference was observed in the proportion of respondents who had heard of CE infection between Central and Eastern (E) region (OR 1.62, CI: 1.13–2.33, and *p* < 0.005). No differences were observed between Northeastern (NE) and E or between NE and Central region (*p* > 0.05). Notably, 91.4% of the respondents from Northeastern region claimed to have seen patients with CE signs compared with 23.4% and 19.5% from Eastern and Central region, respectively (OR 42.88; CI: 21.94–87.44; *p* < 0.001). None knew his/her CE status. The details are shown in Table 2.

The results in Table 2 show that respondents in Northeastern region were nearly five times more likely to have heard about CE than those in Eastern region (OR = 4.9).

### 3.3. Knowledge of Communities about CE Infection according to the Level of Education

Notably, there was no statistical difference in the awareness about CE between the respondents with informal and primary education in Northeastern and Central regions (*p* > 0.05). Similarly, there was no statistical difference in the awareness about CE between the respondents with secondary and tertiary education in all the regions of Uganda (*p* > 0.05%). However, the respondents with secondary and tertiary education were more aware about CE infection than those with informal and primary education in all the regions (*p* < 0.001). The details are as shown in Table 3.

 The findings in Table 3 show that although there was high statistical difference between respondents with low and high level of education in the regions because of the differences in numbers, it is most unlikely that persons with high level of education would be more aware of CE than those with low level of education (OR less than 1).

### 3.4. Knowledge of Communities about CE Infection according to Sex

There was no statistical difference in the awareness about CE between male and female respondents in all the study regions (*p* > 0.05).

### 3.5. Health Workers Knowledge about Echinococcosis

Ninety percent of the health staff from Northeastern region and 96.2% and 93.1% from Eastern and Central regions, respectively, were aware of CE (*p* > 0.05). 57.7 percent of the health staff from Eastern region claimed to have seen patients with CE compared with 80.0% from Northeastern region (*p* < 0.05).

None of the health staffs knew how to screen for CE and knew his/her CE status. The details are shown in Table 4.

Although the results in Table 4 show that there was no statistical difference between health workers in all the study regions as far as their level of knowledge about CE was concerned, respondents from Northeastern region were nearly two times more likely to see tape worm than those from Central region (OR = 2.07).

### 3.6. Attitudes of the Communities towards the Screening and Treatment for Cystic Echinococcosis

32.1% of the community participants from Northeastern region and 35.0% from Central region had a positive attitude towards going to hospital for treatment compared with 60.5% from Eastern region (*p* < 0.001). Twenty percent (19.8%) from Eastern region had positive attitude towards visiting witch doctors for treatment compared to 62.0% and 60.4% of the respondents from Northeastern and Central region, respectively (OR 6.61; CI: 4.81–9.81; *p* < 0.001 and OR 6.18; CI: 4.52–8.48; *p* < 0.001, resp.). The details are shown in Table 5.

The results in Table 5 show that respondents in Northeastern and Central regions were six times more likely to visit witch doctor for CE treatment than those from Eastern region (OR = 6.61 and 6.18, resp.). The results also show that although more respondents from Eastern region preferred hospital treatment for CE to witchcraft than those from Northeastern region, which was statistically significant, the likelihood that respondents from Eastern region would go to hospital was very low (OR less than 1).

## 4. Attitudes of the Health Staff towards the Screening and Treatment for Cystic Echinococcosis

There was no statistical difference between the health staff in all the study regions as far as their attitude towards echinococcosis screening and treatment was concerned (*p* > 0.05).

### 4.1. Beliefs of the Communities about Cystic Echinococcosis

The study showed that 36.7% of the community respondents from Northeastern region and 15.3% from Eastern region believed that drinking raw milk and eating raw meat causes CE (OR 3.3; CI: 1.81–6.16; *p* < 0.00). 43.9% of the respondents from Eastern and 28.7% from Central region believed CE is caused by sharing shelter with animal compared to 11.7% from Northeastern region (*p* < 0.001). Similarly, 31.3% of the respondents from Northeastern region believed CE is caused by witchcraft compared with 14.3% from Eastern region (OR 2.72; CI: 1.46–5.10; *p* < 0.001). Less than 3.4% of the respondents in all the regions believed CE is caused by eating food contaminated by dog fecal. The rest of the details are shown in Table 6.

The results in Table 6 show that respondents in Northeastern region were three times more likely to believe that CE is caused by drinking raw milk than those from Eastern region (OR = 3).

### 4.2. Beliefs of the Health Workers about Cystic Echinococcosis

There was no statistical difference in the beliefs about CE infection between the health workers in all the regions (*p* > 0.05).

### 4.3. Sources of Information of the Communities about Cystic Echinococcosis

Their main sources of information of the communities in all the regions about echinococcosis in descending order were traditional healers, elders in community and health workers, and hospitals/health centers.

### 4.4. Sources of Information of the Health Workers about Cystic Echinococcosis

Their main sources of information of the health staff in all the regions about echinococcosis in descending order were fellow health workers, hospitals/health centers, community, and traditional healers.

## 5. Discussion

This study was conducted to determine the knowledge gaps, beliefs, and attitudes of the communities and health workers about echinococcosis infection in pastoral region of Northeastern and agropastoral regions of Eastern and Central Uganda [12]. There was variability in the awareness, attitudes, and beliefs about CE among the respondents in the study regions. Our study found the pastoral communities in Northeastern communities to be more aware of CE than the agropastoral communities in Eastern and Central regions. However, this finding is not in agreement with the a study by Nyakarahuka et al. [16] which found awareness about CE in pastoral communities of Kasese in Western region to be low. The higher awareness about CE in pastoral communities noted in Northeastern region was probably influenced by the high prevalence of 3.9% of CE among the communities in this region as compared to 1.2% in Eastern and 2.7% in Central region (Othieno et al. [3]). This finding is in agreement with the study by Li et al. [17], which noted that awareness about CE was high in areas that were endemic for CE. This could also be one of the likely reasons why most of the communities in Northeastern region claimed to have seen more persons with CE signs than those from Eastern and Central regions, which is in conformity with the study by Craig et al. [18] which found that communities where the prevalence of CE is high were more likely to come across persons with CE.

Although there was no statistical difference in the knowledge ability about tape worm between respondents from Eastern region and Central region and between Central region and Northeastern region concerned, the likelihood that respondents from Eastern region would know about CE worm would be higher than those from Central region (OR 2.28, *p* < 0.354) (Table 2). Similarly, the likelihood that respondents from Northeastern region would know about CE worm would be higher than those from Central region, respectively (OR 1.19, *p* < 0.878) (Table 2). There was little variation in the way the CE tapeworm was locally called among the respondents who claimed to know CE tape worm. Those from Northeastern and Eastern regions were all calling it* “ecidait”* generally meaning a worm. This is probably because communities from these regions shared the same migration (Okwi et al. [19]). Those respondents from Central region called it* “enfana”* also generally meaning worm.

While the findings of this study (Table 3) are in conformity with the study by Omadang et al. [20] which noted that the level of awareness increased with level of education, this study found that respondents from NE region, with high CE prevalence (Othieno et al. [3]), were more likely to be more aware of CE than those from Eastern and Central regions of low prevalence regardless of their level of education.

Notably, it was found that the difference in the awareness between male and female respondents in all the regions was marginal. This agrees with the study by Omrani et al. [21] which noted there was no statistical significance in the awareness about CE between males and females in the same study population.

Whereas the health respondents in all the study regions were aware that CE can be screened and treated in hospital; surprisingly, none knew how to screen for CE and none had participated in the screening exercise for CE. This probably explains why none of the health workers and community members had been screened for CE and knew his/her CE status in spite of the fact that CE cases are present in these regions (Othieno et al. [3]). Our findings are in agreement with a study by Reyes et al. [10], which found that lack of knowledge of the health workers on the use of ultrasound for detection of CE was a likely major contributor of endemicity of CE since they are not treated. This was also noted by Nasrieh et al. [22] study, which observed that lack of knowledge of the health workers on the use of ultrasound for detection of echinococcosis was probably association with the spread of the disease in the community. A similar study by Dawit et al. [23]** ‎**found lack of understanding about CE detection by health professionals was associated with poor management, control of CE, and high transmission of CE in the communalities, since those with the disease were not being detected and treated. These observations are equally in agreement with a study by John et al. [9] which showed that lack of adequate knowledge by health workers on echinococcosis detection was associated with poor management and high prevalence of echinococcosis in sub-Saharan African countries.

The majority of the community respondents preferred going to witch doctors for treatment for CE. This is probably because none the health staff in these regions knew how to screen for CE (Tables 4 and 5). Our findings tally with the study by Karim [24] which noted that members of the communities were often seeking treatment for CE from traditional healers due to poor provision of health care.

Respondents in the study regions had divergent beliefs about the causes of CE. The majority of the community participants in all the study regions believed CE is caused by drinking raw milk and eating raw meat. Few of the participants believed that CE is punishment from God and is due to witchcraft which was in conformity with the study by Nyakarahuka et al. [16]. While a study by Acosta-Jamett et al. [25, 26] found dog fecal as a risk factor for CE in Chile, most of the community respondents in this study did not believe that eating dog fecal-contaminated food was the key mode of CE transmission. This is in agreement with the findings by El Berbri et al. [27], which showed that most of the respondents had poor beliefs about the role of a dog in CE transmission. The same observation was made by Oba et al. [7] study, which found that most respondents had poor knowledge of CE transmission.

The main sources of information about CE infection among the communities in all the study regions were found to be traditional healers. This probably explains why most of the respondents in Northeastern and Central regions believed that CE is caused by witchcraft and were inclined towards traditional healers (witch doctors) for health services (Table 5).

## 6. Limitations of the Study

The participants were not interviewed from the households because some of these communities especially pastoral communities do not have permanent houses since they continuously move from one place to the other in such pasture. Participants' responses of CE disease were limited to only physical observations of CE signs and thus subjective interpretations of CE could have introduced errors in the study.

## 7. Conclusions and Recommendations

Communities in Northeastern region were more aware of CE than those from Eastern and Central regions, respectively. The majority of the communities in all regions were not aware that CE can be treated in hospital and can be caused by eating food contaminated by dog fecal. None of the health staff was screening for CE and none of the community respondents including health workers had been screened for CE. Sensitizing the communities about CE and its detection and treatment is cardinal to the prevention and control of CE. There is also need to train the health staff preferably radiographers on the use of ultrasound for detection of CE and have these services established at referral health facilities.

## Figures and Tables

**Table 1:** Sociodemographic characteristics (distribution) of respondents.

Variable	Category	Northeastern		Eastern		Central		Totals
		Number	(%)	Number	(%)	Number	(%)	
	Community Respondents	421	34.1	405	32.8	409	33.1	1235
	Health staff	20	26.7	26	34.7	29	38.6	75

Age (years)	Below 18	54	32.1	65	38.7	49	29.2	168
	21 to 40	233	33.5	224	32.2	238	34.2	695
	41 to 60	31	33.6	123	31,5	136	34.9	390
	60 to 80	23	40.4	19	33.3	15	26.3	57

Sex	Male	180	25.0	291	40.4	249	34.6	720
	Female	261	42.2	140	23.7	189	32.0	590

Education level	Informal	291	45.3	167	25.9	185	28.8	643
	Primary	125	24,6	200	39.3	184	36.1	509
	Secondary	20	18.9	36	34.0	50	47.2	106
	Tertiary	5	9.6	28	53.9	19	36.5	52

**Table 2:** Knowledge level of communities about CE infection in humans.

Knowledge attribute	Region	Response				Total	OR	95% CI	*p* value
		Yes (*n*)	(%)	No (*n*)	%				
Heard of CE	Northeastern	256	60.8	165	39.2	421	4.85	3.60−6.60	0.001^*∗∗*^
	Eastern	98	24.2	307	75.8	405			
	Northeastern	256	60.8	165	39.2	421	5.73	4.22−7.82	0.001^*∗∗*^
	Central	87	21.3	322	78.7	409			
	Eastern	98	24.2	307	75.8	405	1.18	0.85−1.64	0.321
	Central	87	21.3	322	78.7	409			

			*Only for those aware of CE*						

Known a CE tapeworm	Northeastern	7	1.7	249	98.3	256	0.52	0.16−1.84	0.295
	Eastern	5	5.1	93	94.9	98			
	Northeastern	7	2.7	249	98.3	256	1.19	0.26−8.53	0.878
	Central	2	2.3	85	97.7	87			
	Eastern	5	5.1	93	94.9	98	2.28	0.44−17.32	0.354
	Central	2	2.3	85	97.7	87			

Had seen patients with CE signs	Northeastern	234	91.4	22	8.6	256	34.06	18.22−65.94	0.001^*∗∗*^
	Eastern	23	23.5	75	76.5	98			
	Northeastern	234	91.4	22	8.6	256	42.88	21.94−7.44	0.001^*∗∗*^
	Central	17	19.5	70	80.5	87			
	Eastern	23	23.4	75	76.5	98	1.44	0.71−2.95	0.312
	Central	17	19.5	70	80.5	87			

Only for those aware									

Know their CE status	Northeastern	None							
	Eastern	None							
	Central	None							

NS = *p* > 0.05 not significant, *p* < 0.05 significant, ^*∗∗*^*p* < 0.01 highly significant, and *p* < 0.001 very highly significant. OR = odds ratio and CI = confidence interval.

**Table 3:** Knowledge of communities about CE infection according to the level of education.

Region	Number	Heard about CE				OR	95% CI	*p* value
		Yes	(%)	No	(%)			
*Northeastern (421)*								
Informal	278	123	44.2	155	55.8	0.78	0.44–1.34	0.355
Primary	119	60	50.4	59	49.6			
Informal	278	123	44.2	155	55.8	0.27	0.14–0.50	0.001
Secondary	19	15	78.9	4	21.1			
Informal	278	123	44.2	255	55.8	0.20	0.10–0.37	0.001
Tertiary	5	4	80.0	1	20.0			
Primary	119	60	50.4	59	49.6	0.21	0.11–0.39	0.001
Secondary	19	15	78.9	4	21.1			
Primary	119	60	50.4	59	49.6	0.26	0.13–0.48	0.001
Tertiary	5	4	80.0	1	20.0			
Secondary	19	15	78.9	4	21.1	0.94	0.47–1.87	0.850
Tertiary	5	4	(80.0)	1	20.0			
*Central (409)*								
Informal	173	18	10.4	155	89.6	0.66	0.27–1.57	0.353
Primary	172	24	14.0	148	86.0			
Informal	173	18	10.4	155	89.6	0.04	0.03–0.12	0.001
Secondary	46	34	73.9	12	26.1			
Informal	173	18	10.4	155	89.6	0.03	0.01–0.11	0.001
Tertiary	18	14	77.8	2	22.2			
Primary	172	24	14.0	148	86.0	0.06	0.05–0.19	0.001
Secondary	46	34	73.9	12	26.1			
Primary	172	24	14.0	148	86.0	0.03	0.01–0.15	0.001
Tertiary	18	14	77.8	2	22.2			
Secondary	46	34	73.9	12	26.1	0.79	0.41–1.52	0.484
Tertiary	18	14	77.8	4	22.2			
*Eastern* (*n* = 405)	405							
Informal	157	16	10.2	141	89.8	0.43	0.20–0.92	0.001
Primary	188	35	18.6	153	81.4			
Informal	157	16	10.2	141	89.8	0.03	0.01–0.08	0.001
Secondary	34	26	76.5	8	23.5			
Informal	157	16	10.2	141	89.8	0.01	0.006–0.04	0.001
Tertiary	26	23	88.5	2	11.5			
Primary	188	35	18.6	153	81.4	0.07	0.04–.15	0.001
Secondary	34	26	76.5	8	23.5			
Primary	188	35	18.6	153	81.4	0.03	0.01–0.06	0.001
Tertiary	26	23	88.5	3	11.5			
Secondary	34	26	76.5	8	23.5	0.69	0.29–1.59	0.389
Tertiary	26	23	88.5	3	11.5			

NS = *p* > 0.05 not significant, *p* < 0.05 significant, *p* < 0.01 highly significant, and *p* < 0.001 very highly significant. OR = odds ratio; CI = csonfidence interval.

**Table 4:** Knowledge of the level of the health workers about CE infection.

Knowledge attribute	Region	Response		Totals	OR (95% CI)	*p* value
		Yes, *n* (%)	No, *n* (%)			
Heard of CE	Northeastern	18 (90.0)	2 (10.0)	20	0.37 (0.12−5.15)	0.238
	Eastern	25 (96.0)	1 (4.0)	26		
	Northeastern	18 (90.0)	2 (10.0)	20	0.67 (0.07−6.95)	0.361
	Central	27 (93.1)	2 (6.9)	29		
	Eastern	25 (96.2)	1 (3.8)	26	1.83 (0.13−56.67)	0.341
	Central	27 (93.1)	2 (6.9)	29		

Know a CE Tapeworm	Northeastern	15 (75.0)	5 (25.0)	20	0.69 (1.11−4.49)	0.295
	Eastern	22 (84.6)	4 (15.4)	26		
	Northeastern	15 (75.0)	5 (25.0)	20	1.42 (0.30−7.92)	0.340
	Central	21 (72.4)	8 (27.6)	29		
	Eastern	22 (84.6)	4 (15.4)	26	2.07 (0.45−11.27)	0.354
	Central	21 (72.4)	8 (27.6)	29		

Had seen patients with CE signs	Northeastern	16 (80.0)	4 (20.0)	20	0.19 (0.02−0.96)	0.044^*∗*^
	Eastern	15 (57.7)	11 (42.3)	26		
	Northeastern	16 (80.0)	4 (20.0)	20	1.80 (0.31−14.82)	0.273
	Central	22 (75.9)	7 (24.1)	29		
	Eastern	15 (57.7)	11 (42.3)	26	0.35 (0.09−1.22)	0.051
	Central	22 (75.9)	7 (24.1)	29		

Only for those aware of CE						

Know how to screen for CE	Northeastern	None				
	Eastern	None				
	Central	None				
Know their CE status	Northeastern	None				
	Eastern	None				
	Central	None				

NS = *p* > 0.05 not significant, ^*∗*^*p* < 0.05 significant, OR = odds ratio, and CI = confidence interval.

**Table 5:** Attitudes of the communities towards screening and treatment for CE infection.

Attitude attribute	Region		Response			Totals	OR	95% CI	*p* value
		Yes (*n*)	%	No (*n*)	%				
Willingness to be screened *(only those aware of CE)*	Northeastern	182	71.1	74	28.9	256	0.98	0.58−1.68	0.477
	Eastern	70	71.1	28	28.6	98			
	Northeastern	182	71.1	74	28.9	256	0.73	0.14−1.29	0.146
	Central	67	77.0	20	23.0	87			
	Eastern	70	71.1	28	28.6	98	0.73	0.51−1.29	0.146
	Central	67	77.0	20	23.0	87			

Prefer hospital treatment	Northeastern	135	32.1	286	67.9	421	0.31	0.23−0.41	0.001
	Eastern	245	60.5	160	40.0	405			
	Northeastern	135	32.1	286	67.9	421	0.89	0.66−1.17	0.189
	Central	143	35.0	266	65.0	409			
	Eastern	245	60.5	160	40.0	405	2.85	2.14−3.79	0.001
	Central	143	35.0	266	65.0	409			

Go to witch doctors	Northeastern	261	62.2	160	38.0	421	6.61	4.81−9.08	0.001
	Eastern	80	19.8	325	80.2	405			
	Northeastern	261	62.0	160	38.0	421	1.07	0.81−1.47	0.318
	Central	247	60.4	162	39.6	409			
	Eastern	80	19.8	325	80.2	405	6.18	4.52−8.48	0.001
	Central	247	60.4	162	39.6	409			

NS = *p* > 0.05 not significant and *p* < 0.001 very highly significant. OR = odds ratio and CI = confidence interval.

**Table 6:** Beliefs of the communities about cystic echinococcosis.

Belief attributes	Regional comparison		Response			OR	95% CI	*p* value
		Yes (*n*)	%	No (*n*)	%			
*Only those aware of CE* CE is caused by punishment from God	Northeastern	96	37.5	160	62.5	1.85	1.10−3.17	0.019
	Eastern	24	24.5	74	75,5			
	Northeastern	96	37.5	160	62.5	1.14	0.69−1.91	0.620
	Central	30	34.5	57	65.5			
	Eastern	24	24.5	74	75.5	0.51	0.26−0.98	0.043
	Central	30	34.5	57	65.5			

CE is caused by drinking raw milk and eating raw meat.	Northeastern	94	36.7	162	63.3	3.3	1.81−6.16	0.001
	Eastern	15	15.3	83	84.7			
	Northeastern	94	36.7	162	63.3	1.05	0.63−1.75	0.861
	Central	31	35.6	56	64.4			
	Eastern	15	15.3	83	84.7	0.32	0.15−0.66	0.001
	Central	31	35.6	56	64.4			

CE is caused by witch craft	Northeastern	80	31.3	176	68.7	2.72	1.46−5.10	0.001
	Eastern	14	14.3	84	85.7			
	Northeastern	80	31.3	176	68.7	0.43	0.26−0.70	0.001
	Central	45	51.7	42	48.3			
	Eastern	14	14.3	84	85.7	0.16	0.08−0.31	0.001
	Central	45	51.7	42	48.3			

CE is caused by sharing shelter with animal	Northeastern	30	11.7	226	88.3	0.17	0.10−0.30	0.001
	Eastern	43	43.9	55	56.1			
	Northeastern	30	11.7	226	88.3	0.33	0.18−0.61	0.001
	Central	25	28.7	62	71.3			
	Eastern	43	43.9	55	56.1	1.93	1.05−3.60	0.034
	Central	25	28.7	62	71.3			

CE is caused by eating food contamination by dog fecal	Northeastern	6	2.3	250	97.7	1.15	0.24−8.41	0.912
	Eastern	2	2.0	96	98.0			
	Northeastern	6	2.3	250	97.7	0.67	0.16−3.36	0.597
	Central	3	3.4	84	96.6			
	Eastern	2	2.0	96	98.0	0.59	0.10−3.58	0.593
	Central	3	3.4	84	96.6			

NS = *p* > 0.05 not significant, *p* < 0.05 significant, *p* < 0.01 highly significant, and *p* < 0.001 very highly significant. OR = odds ratio; CI = confidence interval.

## Data Availability

Data is available in hard copies and can be accessed on request.

## Abbreviations

MoH: Ministry of Health
ERC: Ethics and Research Committee
MUCVM: Makerere University College of Veterinary Medicine
EC: Ethical Council
DHO: District Health Officer
CAO: Chief Administrative Officer
DVO: District Veterinary Officer
LCs: Local Councils.
